# Prehospital Volume Therapy as an Independent Risk Factor after Trauma

**DOI:** 10.1155/2015/354367

**Published:** 2015-04-09

**Authors:** Bjoern Hussmann, Matthias Heuer, Rolf Lefering, Alexander Touma, Carsten Schoeneberg, Judith Keitel, Sven Lendemans

**Affiliations:** ^1^Trauma Surgery Department, Alfried Krupp Hospital Essen, Alfried-Krupp-Straße 21, 45131 Essen, Germany; ^2^Surgery Department, Philippusstift Essen, Huelsmannstraße 17, 45355 Essen, Germany; ^3^Institute for Research in Operative Medicine (IFOM), Witten/Herdecke University, Ostmerheimer Straße 200, 51109 Cologne, Germany; ^4^Trauma Surgery Department, University Hospital Essen, Hufelandstraße 55, 45147 Essen, Germany

## Abstract

*Background*. Prehospital volume therapy remains widely used after trauma, while evidence regarding its disadvantages is growing. The primary objective of this study was to investigate the volume administered in a prehospital setting as an independent risk factor for mortality.* Material and Methods*. Patients who met the following criteria were analyzed retrospectively: Injury Severity Score = 16, primary admission (between 2002 and 2010), and age = 16 years. The following data had to be available: volume administered (including packed red cells), blood pressure, Glasgow Coma Scale, therapeutic measures, and laboratory results. Following a univariate analysis, independent risk factors for mortality after trauma were investigated using a multivariate regression analysis.* Results*. A collective of 7,641 patients met the inclusion criteria, showing that increasing volumes administered in a prehospital setting were an independent risk factor for mortality (odds ratio: 1.34). This tendency was even more pronounced in patients without severe traumatic brain injury (TBI) (odds ratio: 2.71), while the opposite tendency was observed in patients with TBI.* Conclusions*. Prehospital volume therapy in patients without severe TBI represents an independent risk factor for mortality. In such cases, respiratory and circulatory conditions should be stabilized during permissive hypotension, and patient transfer should not be delayed.

## 1. Introduction

For most severely injured patients, prehospital volume therapy is a measure for maintaining tissue and organ perfusion. In such patients, uncontrollable bleeding following trauma is still considered the most common preventable cause of death [1–4]. The immediate effects of bleeding and shock may result in direct and indirect sequelae in surviving patients. For example, 20% of patients develop multiorgan failure during hospitalisation and 20% experience septic episodes. Multiorgan failure and septic conditions, beside thromboembolic complications, lead to a significant increase of mortality following severe trauma [5]. Hence, hemorrhagic shock and its consequences are the second most common cause of death, with severe traumatic brain injury (TBI) being number one [6].

The options for the prehospital treatment of hemorrhagic shock are limited. In addition to stopping the bleeding, that is, the hemostasis of externally visible bleeding via compression, in accordance with the Advanced Trauma Life Support (ATLS) guidelines, volume therapy is of paramount importance [7]. In the recent literature, the excessive nonindicated use of volume substitution in patients with severe trauma has been increasingly questioned. In the late 1990s, Bickell showed that rapid transfer and modest volume therapy (accepting permissive hypotension) appeared to be useful for patients with penetrating trauma [8–10]. Restricted volume therapy also increasingly appears to be useful in patients with blunt trauma and hemorrhagic shock [11–15]. Publications from our group have demonstrated that extensive volume therapy is associated with an increase in mortality, even in children [16–19]. These studies showed that the patients' coagulation status was impaired, and the authors concluded that this was attributable to a “dilutive effect” of excessive volume therapy. In a recent study from USA, Haut et al. showed that extensive volume therapy is associated with worsened outcomes. The authors concluded that prehospital volume therapy is no longer useful [20]. According to the current literature, the number of other therapies performed at the accident site has increased along with the increased use of volume therapy, leading to extended emergency treatment times and, as a consequence, longer delays in the patient's admission to the hospital [17, 18, 21, 22]. In this context, Clarke et al. demonstrated that mortality increased by 1% for every three minutes without emergency surgery in patients with abdominal trauma [23].

The supporters of extensive volume therapy justify its use by focusing on the importance of elevating the mean arterial blood pressure and maintaining sufficient organ perfusion [24]. Moreover, it remains difficult to justify permissive hypotension in patients with simultaneous severe TBI. According to the recent literature, normotension should be targeted to maintain a sufficient level of cerebral perfusion pressure [25].

A search of the current literature raises the question of whether the volume and number of substitutions have consequences for hemorrhagic shock during the posttraumatic course. Thus, the hypothesis of this study was that extensive prehospital volume replacement has a negative impact on patient mortality and represents an independent risk factor.

## 2. Material and Methods

The TraumaRegister DGU of the German Trauma Society (Deutsche Gesellschaft für Unfallchirurgie, DGU) was founded in 1993. The aim of this multicentre database is an anonymous and standardized documentation of severely injured patients.

Data are collected prospectively in four consecutive time phases from the site of the accident until discharge from hospital: (A) prehospital phase, (B) emergency room and initial surgery, (C) intensive care unit, and (D) Discharge. The documentation includes detailed information on demographics, injury pattern, comorbidities, pre- and in-hospital management, course on intensive care unit, relevant laboratory findings including data on transfusion, and outcome of each individual. The inclusion criterion is admission to hospital via emergency room with subsequent ICU/ICM care or reaching the hospital with vital signs and death before admission to ICU. The infrastructure for documentation, data management, and data analysis is provided by AUC, Academy for Trauma Surgery (AUC, Akademie der Unfallchirurgie GmbH), a company affiliated to the German Trauma Society. The scientific leadership is provided by the Committee on Emergency Medicine, Intensive Care and Trauma Management (Sektion NIS) of the German Trauma Society. The participating hospitals submit their data anonymously into a central database via a web-based application. Scientific data analysis is approved according to a peer review procedure established by Sektion NIS. The participating hospitals are primarily located in Germany (90%), but a rising number of hospitals of other countries contribute data as well (at the moment from Austria, Belgium, China, Finland, Luxembourg, Slovenia, Switzerland, Netherlands, and UAE). Currently, approximately 25.000 cases from more than 600 hospitals are entered into the database per year. Participation in TraumaRegister DGU is voluntary. For hospitals associated with TraumaNetzwerk DGU, however, the entry of at least a basic data set is obligatory for reasons of quality assurance.

The present study is in line with the publication guidelines of the TraumaRegister DGU (TR-DGU) and registered as TR-DGU project ID 2012-002.

Only patients from Germany and Austria were included in this study to minimize variations related to the use of different rescue systems. All of the patients were attended by a physician prior to hospital admission.

Sepsis was defined according to the American College of Chest Physicians/Society of Critical Care Medicine (ACCP-SCCM) consensus conference definition [26]. Single organ failure was defined as a Sequential Organ Failure Assessment (SOFA) score ≥3 [27]. The hospitals participating in the TraumaRegister DGU entered the SOFA score as the total value in the registry; therefore, no conclusions about individual patient management or intervention could be drawn. Multiple organ failure (MOF) was listed if simultaneous organ failure was recorded for at least two organs. Prehospital parameters, length of hospital stay, and coagulation status were examined separately for each group. To determine coagulation, we used the prothrombin ratio, a parameter that is commonly used in Germany and that corresponds to the International Normalized Ratio (INR).

Patients seen between 2002 and 2010 were selected for this study according to the following criteria:

1. Primary admission to the hospital (no transfers).
2. Injury Severity Score (ISS) ≥16.
3. Age ≥16 years.
4. Data available for prehospital and hospital volume therapy and packed red blood cell administration, Glasgow Coma Scale (GCS), hemoglobin concentration, base excess, one coagulation parameter (e.g., prothrombin time), blood pressure at the accident site, blunt trauma, therapeutic measures (resuscitation, intubation, insertion of chest tube), and prehospital time.

In this study, 7,641 cases met these criteria and were further investigated.

The subsequent analysis was conducted in two steps:

1. Assignment to one of 5 groups and univariate analysis based on the volumes administered in the prehospital setting:
  - Group 1: 0–500 mL.
  - Group 2: 501–1000 mL.
  - Group 3: 1001–1500 mL.
  - Group 4: 1501–2000 mL.
  - Group 5: ≥ 2001 mL.
2. Multivariate regression analysis with the dependent characteristic “mortality” in different subgroups (see step 1). The variables included in the stepwise regression were as follows: prehospital volume replacement, in-hospital volume replacement, age, Revised Trauma Score, blood pressure at the accident site, ISS, New-ISS, AIS (head, thorax, abdomen, and extremities, including pelvis), blunt trauma, penetrating trauma, resuscitation at the accident site, time from accident to hospital admission, prehospital intubation, prehospital chest tube, base excess at admission, hemoglobin concentration at admission, cause of accident, prothrombin time in hospital, and prehospital catecholamines.

A subgroup analysis differentiating patients with severe TBI (AIS head ≥ 4 [*n* = 3187]) from those* without* severe TBI (AIS head < 4 [*n* = 4454]) was conducted under the same conditions used to differentiate the total population using a multivariate regression analysis.

### 2.1. Statistics

The Statistical Package for the Social Sciences (SPSS; version 17, Chicago, IL, USA) was used. The data were analyzed univariately using Student's *t*-test for continuous variables and the *χ* ^2^ test for categorical variables. The results are expressed as the mean ± standard deviation for continuous values and as percentages for categorical variables.

A multivariate analysis was performed with a stepwise logistical regression analysis. Mortality was used as a dependent variable to identify the risk factors for mortality after trauma, and the results are expressed as odds ratios (OR) and 95% confidence intervals. We applied a significance level *α* of 5% to all of the statistical tests.

## 3. Results

### 3.1. Univariate Analysis

The proportion of male patients significantly increased with increases in the prehospital volume (group 1: 69.9% and group 5: 76.9%; *P* ≤ 0.001), while age declined with increasing prehospital volume administration. As expected, most of the injuries were blunt trauma injuries (96.0%). Both the AIS of the individual body regions (except AIS head) and the ISS increased with the administered volume (ISS: group 1: 26.8% and group 5: 33.2%; *P* ≤ 0.001; Table 1).

Regarding the accident causes, the proportion of car and motorcycle accidents increased significantly with increasing volumes. The opposite trend was observed for low and high falls (Table 2).

Prehospital measures such as intubation, resuscitation, catecholamine administration, chest tube insertion, and the prehospital emergency treatment times significantly increased with increases in volume, as Tables 3(a) and 3(b) show.

The clinical patient parameters in Tables 3(a) and 3(b) indicate a worsening tendency among patients when the administered volume increased (base excess: group 1: −2.0 ± 4.0 and group 5: −5.2 ± 5.3; *P* ≤ 0.001; prothrombin time: group 1: 84.7% and group 5: 62.5%; *P* ≤ 0.001). Similarly, the number of mass transfusions declined with extended prehospital volume therapy (group 1: 2.2% and group 2: 24.7%; *P* ≤ 0.001).

Regarding the outcome parameters and mortality, an increase can be observed with increasing volumes (mortality: group 1: 18.3% and group 5: 24.0%; *P* ≤ 0.001; Table 4).

### 3.2. Multivariate Regression Analysis

#### 3.2.1. Multivariate Regression Analysis of the Total Population (*n* = 7641)

The risk of death from severe trauma significantly increased with older age (Table 5). The parameters assessed with the Revised Trauma Score (RTS), such as GCS, systolic blood pressure, and respiratory rate at the accident site, also show that a worse GCS score was associated with an increased risk of death. Severe head injuries (AIS ≥4) were also significantly associated with an increased mortality risk (Table 5).

In addition to the prehospital measures listed in Table 5, the volume administered in the prehospital setting increases the mortality, beginning with volumes >1500 mL.

#### 3.2.2. Multivariate Regression Analysis of Patients without Severe TBI (*n* = 4454)

In the subgroup without severe TBI, the increased likelihood of death associated with the volume administered in a prehospital setting was particularly striking (odds ratio of death 501–1000 mL: 1.44, odds ratio of death ≥ 2001: 2.71).

The remaining parameters showed the same tendencies as in the total population (Table 6).

#### 3.2.3. Multivariate Regression Analysis of Patients with Severe TBI (*n* = 3187)

The trend that was observed in the subgroup without severe TBI was not identified for the subgroup with severe TBI, as Table 7 shows. In fact, volume therapy administered in a prehospital setting had protective effects in the patients with severe TBI, particularly when the patients received a volume of 1001–2000 mL. Again, this effect is reduced with increasing volumes, and it was not observed in patients who received volumes of more than 2001 mL.

The remaining parameters showed the same tendencies as in the total population (Table 7).

## 4. Discussion

Our study clearly shows that extended volume therapy in severely injured patients without severe traumatic brain injury (AIS ≥4) can be considered an independent risk factor for mortality. Because this study was a retrospective analysis, it cannot provide answers about why prehospital volume therapy increased mortality. Based on the results of this study and on the current literature, factors such as the dilution of coagulation factors in the blood (which becomes evident when observing the prothrombin times in this study), the dissolution of clotting factors, and the active maintenance of hemorrhage by increasing blood pressure can be discussed [16–18, 28–31]. In this context, prehospital volume administration, beside an existing trauma-related consumption of clotting factors, possibly represents an additional factor that promotes bleeding. Furthermore, volume therapy at the accident site despite normal blood pressure would be another factor that may increase bleeding tendency. Due to the retrospective study design with anonymised patients, it is not possible to conclusively clarify the level of impact in each individual patient. Nevertheless, these factors play a crucial role, particularly in patients with blunt trauma and uncontrollable internal bleeding (e.g., splenic rupture). The use of permissive hypotension until the patients receive definite care in the hospital (e.g., surgery or embolization), as Bickell et al. and other authors have proposed for patients with penetrating and blunt trauma, respectively, appears useful in this context [8–10, 32].

Furthermore, extended prehospital volume therapy is associated with prolonged emergency treatment times. This relationship has also been reflected in the recent literature and is commonly fatal in severely injured, bleeding patients because life-saving surgical procedures that can only be performed in the hospital are delayed when prehospital volume therapy is administered. In this context, US studies demonstrated that patients who had been transferred to the hospital in a private car were admitted earlier and had higher survival rates after penetrating trauma compared with patients who had been treated and transferred by EMS professionals [33, 34]. Based on the current literature, these are exceptional cases, and transfer in a private car cannot be recommended as a general rule. Based on this retrospective study, step 1 of the univariate analysis cannot manage to clarify for each individual patient why more severely injured patients had longer emergency treatment times and enhanced levels of prehospital volume therapy. The principal guideline should be as follows: the more severe the injury is the sooner the patient should be transferred to the hospital. This particularly applies to patients with active bleeding. In this context, a recently published prospective randomised study has shown again that time to surgical intervention is longer than one hour, particularly after blunt trauma. Thus, the golden hour of shock is not achieved in reality [35]. The objective of the univariate analysis was to demonstrate principal relations between prehospital volume therapy and parameters such as outcome. Interestingly, the haemoglobin value upon admission as a sign for bleeding did not represent an independent risk factor in the multivariate analysis. In terms of this aspect, one should consider that haemoglobin value and prehospital volume are correlating closely. It is inevitable that prospective randomised studies will be conducted in this context. The heterogeneous prehospital patient population is certainly one reason why only one prospective study can be found in the literature [36].

Moreover, the remaining parameters, such as age (one of the most influential factors following severe trauma), must also be considered when examining independent risk factors for mortality after severe trauma. While the age factor cannot be improved with prehospital therapies, it will represent a great challenge in the future given the increasing elderly population. Comorbidities such as coronary heart disease and the associated use of anticoagulants imply additional patient care efforts and aspects to consider after severe trauma.

Based on data from the US National Trauma Data Bank (NTDB), Haut et al. demonstrated in a multivariate analysis that extended volume substitution after severe trauma increases mortality. Those authors concluded that this approach should no longer be used [20]. However, the study by Haut et al. does not differentiate between different volume levels. The study investigated volume substitution versus no volume administration at all. Most of the patients in this study had an ISS <9 (47.1%), and it is likely that volume substitution was not relevant for this patient population. Only 2.5% of the patients had severe TBI, which was something of an underrepresentation. Finally, although the difference in mortality rates was highly significant because of the size of the group, it was only 0.3%. Nevertheless, those results reflect the current literature and are similar to the results of our study.

The use of prehospital volume substitution in severely injured patients with severe traumatic brain injury remains particularly controversial in the literature. Primary brain damage has already occurred during the traumatic event and is difficult to treat in a prehospital setting (e.g., using 30° semirecumbent body positioning). Surgical interventions that are commonly necessary cannot be performed at the accident site. Therefore, prehospital volume therapy is mandatory to prevent secondary damage. In this context, studies have demonstrated that even a short hypotensive phase and the occurrence of a second hit may have detrimental effects on the brain [25, 37]. Therefore, prehospital therapy commonly focuses on volume administration to increase the mean arterial blood pressure and thus improve cerebral perfusion pressure. However, even this approach has been questioned in the literature. Studies have demonstrated that this approach may also lead to the deterioration of cerebral perfusion, for example, by maintaining hemorrhage [38].

Hence, an important result of our study demonstrates that modest prehospital volume therapy (up to 2000 mL) in patients with severe TBI (AIS ≥4) may result in improved mortality, whereas volume therapy in patients without TBI did not show any benefit and even increased the mortality risk. In a retrospective analysis, it is only possible to demonstrate potential relationships; final evaluations are not possible. Nevertheless, maintaining the cerebral perfusion pressure, as Tan et al. propose in their study, appears to be of crucial importance [25]. One limitation to this approach is that it is hardly possible for emergency medical service (EMS) team members at the accident site to evaluate whether every patient has suffered severe TBI with an AIS score ≥4. The GCS may offer some help. Therefore, the decision to provide extended volume therapy at the accident site must be made on a case-by-case basis. A comprehensive standard protocol cannot be established for this situation.

However, this study supports the idea that recommendations for the prehospital treatment of patients with penetrating trauma also apply to patients with blunt trauma. These recommendations include limiting prehospital therapy to the stabilization of the cardiovascular and pulmonary systems and prioritizing rapid transport to a level one trauma center [32].

### 4.1. Limitations

1. Regarding the analysis of the coagulation status, it must be noted that the prothrombin ratio, prothrombin time, and platelet counts are the only parameters that are documented in the TraumaRegister DGU and are available for analysis. Other laboratory values that might be of interest for coagulation (e.g., fibrinogen and protein C) are not documented in TraumaRegister DGU.
2. A retrospective analysis based on anonymized data cannot clarify the individual decisions that were made by the respective EMS team members at the accident site. Furthermore, because of anonymity, the patient files cannot be accessed for additional analyses.

Finally, because we only conducted a retrospective analysis, only associations (no causalities) can be ascribed to the given data. In the future, a prospective randomized study will be indispensable for clarifying the advantages or disadvantages of a particular volume therapy for the most severely injured patients at accident sites.

## 5. Conclusions

Prehospital volume therapy in patients without severe traumatic brain injury represents an independent risk factor for mortality. In such cases, respiratory and circulatory conditions should be stabilized and permissive hypotension should be accepted, and patient transfer should not be delayed. In patients with severe traumatic brain injury, modest prehospital volume therapy can have protective effects.

## Figures and Tables

**Table 1:** Demographic and clinical data of severely injured patients treated prior to hospitalisation with volume fluid replacement therapy.

Group	1 0–500 mL	2 501–1000 mL	3 1001–1500 mL	4 1501–2000 mL	5 ≥2001 mL	Total	*P*
Male (%)	69.9	72.1	71.4	74.2	76.9	72.6	<0.001
Age (years, mean, and SD)	52.6 ± 20.3	46.3 ± 19.7	43.4 ± 19.1	40.1 ± 18.1	39.9 ± 17.3	45 ± 19.7	<0.001
Blunt trauma (%)	97.1	95.9	95.8	95.9	95.3	96	<0.001
GCS ≤ 8 (%)	27.8	33.2	38.1	42.1	42	35.9	<0.001
AIS head ≥ 3 (%)	62.5	55.5	57.5	57.3	53.3	57.3	<0.001
AIS thorax ≥ 3 (%)	47.3	57.4	62.9	64	70.5	59.6	<0.001
AIS abdomen ≥ 3 (%)	15.1	21.1	22.9	24	30	22.2	<0.001
AIS extremities including pelvis ≥ 3 (%)	24.7	34.1	41.8	48.6	58.1	40	<0.001
ISS (mean, SD)	26.8 ± 10.9	28.6 ± 12	30.1 ± 12.2	31.5 ± 13.3	33.2 ± 13.4	29.7 ± 12.5	<0.001
NISS (mean, SD)	34.4 ± 14.6	35.4 ± 14.9	35.9 ± 14.1	37.1 ± 14.9	38.6 ± 14.7	36.1 ± 14.7	<0.001

Values shown as mean, standard deviation (SD), or % of the group. AIS: Abbreviated Injury Scale. ISS: Injury Severity Score. NISS: New Injury Severity Score.

**Table 2:** Cause of injuries volume groups.

Group	1 0–500 mL	2 501–1000 mL	3 1001–1500 mL	4 1501–2000 mL	5 ≥2001 mL	Total	*P*
Car (%)	17	27.5	37.2	39.4	49	32.8	<0.001
Motorcycle (%)	10.3	14.4	16.1	18.3	20.1	15.5	<0.001
Bicycle (%)	9.5	7.8	6.8	5	3.9	6.9	<0.001
Pedestrian (%)	9.4	9.6	8	8.9	5.9	8.5	<0.001
Fall > 3 m (%)	20.8	21.2	18.1	15.8	11.7	18	<0.001
Fall < 3 m (%)	22.1	8.9	4.6	2.5	1.5	8.5	<0.001
Others (%)	10.9	10.4	9.3	10.2	8	9.8	<0.001

Values shown as % of the group.

**(a):** 

Group	1 0–500 mL	2 501–1000 mL	3 1001–1500 mL	4 1501–2000 mL	5 ≥2001 mL	Total	*P*
Prehospital intubation (%)	33.3	53.8	70.6	82.1	91.1	63.6	<0.001
Prehospital resuscitation (CPR) (%)	2.2	2.8	2.8	3.2	5.7	3.2	<0.001
Prehospital catecholamines (%)	3.8	8	10.6	14.6	19.7	10.6	<0.001
Prehospital chest tube (%)	1.1	4.1	6.7	10.2	18.3	7.3	<0.001
Prehospital emergency treatment time, min. (mean, SD)	61 ± 28.3	66.9 ± 27.5	72.2 ± 28.7	75.3 ± 28.8	82.1 ± 31.2	70.6 ± 29.6	<0.001

Values are shown as mean, standard deviation (SD), or % of the group. CPR: cardiopulmonary resuscitation.

**(b):** 

Group	1 0–500 mL	2 501–1000 mL	3 1001–1500 mL	4 1501–2000 mL	5 ≥2001 mL	Total	*P*
Prehospital blood pressure, mmHg (mean, SD)	133.8 ± 34.5	122 ± 34.7	115.4 ± 32.9	109.7 ± 32.3	100.3 ± 34.4	117.6 ± 35.6	<0.001
Blood pressure at admission, mmHg (mean, SD)	130.3 ± 29.7	124.2 ± 29.8	117.8 ± 30.2	116.4 ± 29.5	110.6 ± 31.2	120.7 ± 30.8	<0.001
Prehospital pulse rate per min. (mean, SD)	88 ± 20.6	91.8 ± 23.7	93.8 ± 24.3	97.3 ± 25.3	100.2 ± 28.8	93.7 ± 24.8	<0.001
Pulse at admission per min. (mean, SD)	86.1 ± 19.5	89.3 ± 20.8	89.3 ± 21.6	92.9 ± 22.1	95.6 ± 24.1	90.3 ± 21.8	<0.001
Base Excess at admission (mean, SD)	−2 ± 4	−2.8 ± 4.7	−3.6 ± 4.6	−4.3 ± 4.8	−5.2 ± 5.3	−3.4 ± 4.8	<0.001
Prothrombin time in hospital, sec. (mean, SD)	31.9 ± 16.3	32.5 ± 16.8	34.6 ± 17.2	38.4 ± 23	46.7 ± 31.6	35.8 ± 21.3	<0.001
Prothrombin ratio % (mean, SD)	84.7 ± 23	81.3 ± 21.2	75.8 ± 21.6	70.9 ± 22.6	62.5 ± 23.8	76.1 ± 23.6	<0.001
No units of pRBC (%) in hospital	83	73	62.7	53.2	37.3	63.9	<0.001
1–9 units of pRBC (%) in hospital	14.8	21.4	28.8	33.8	38.1	26.3	<0.001
Massive transfusions ≥10 units of pRBC (%) in hospital	2.2	5.7	8.5	13	24.7	9.9	<0.001

Values are shown as mean, standard deviation (SD), or % of the group. BP: blood pressure; RR: respiratory rate; pRBC: packed red blood cells; CPR: cardiopulmonary resuscitation.

**Table 4:** Clinical course and outcome of patients receiving volume prehospital replacement therapy after trauma.

Group	1 0–500 mL	2 501–1000 mL	3 1001–1500 mL	4 1501–2000 mL	5 ≥2001 mL	Total	*P*
Days of intubation (mean, SD)	6.6 ± 10.8	7.8 ± 11.7	9.3 ± 13.3	10.2 ± 12.7	11 ± 13.1	8.8 ± 12.3	<0.001
Days in ICU (mean, SD)	10.9 ± 13	12.1 ± 13.5	13.7 ± 15.5	14.7 ± 14.2	15.3 ± 15	13.1 ± 14.3	<0.001
Days in hospital (mean, SD)	21.9 ± 20.1	25.6 ± 23.4	27.8 ± 25.7	28.5 ± 25.9	29.7 ± 27.2	26.4 ± 24.5	<0.001
Sepsis (%)	8.6	8.9	10.8	13.7	14.6	11	<0.001
Organ failure (%)	46.5	49.4	56.1	58.1	61.3	53.5	<0.001
Multiorgan failure (%)	29.4	31.5	37.3	40.9	43.3	35.7	<0.001
Death (%)	18.3	16.8	16.9	18.7	24	18.7	<0.001
Death within initial 24 h (%)	7.2	7.7	8.9	10.7	13.4	9.3	<0.001

Values shown as mean, standard deviation (SD), or % of the group. ICU: intensive care unit.

**Table 5:** Multivariate regressions analysis in patients after severe trauma.

	*n*	Odds ratio	95% CI
Age, y			
0–54	5262	Reference	
55–64	826	1.88	1.48–2.39
65–74	818	4.22	3.41–5.25
≥75	735	11.76	9.44–14.65
Revised Trauma Score			
GCS 13–15	3778	Reference	
GCS 9–12	1120	1.46	1.15–1.87
GCS 8–6	901	1.80	1.39–2.34
GCS 5-4	407	3.14	2.29–4.30
GCS 3	1435	4.35	3.44–5.52
Prehospital blood pressure, mmHg			
≥91	5956	Reference	
61–90	1306	1.40	1.17–1.68
0–60	379	2.48	1.83–3.38
Blunt trauma	7337	Reference	
Penetrating trauma	304	1.63	1.14–2.35
Prehospital intubation	4856	1.46	1.16–1.83
Prehospital catecholamines	812	1.54	1.24–1.92
Prehospital resuscitation	248	1.81	1.22–2.68
Prehospital chest tube	561	0.87	0.67–1.14
NISS		1.06	1.05–1.06
AIS head ≥ 4	3187	1.41	1.17–1.68
Prehospital volume, mL			
0–500	1597	Reference	
501–1000	2047	0.91	0.73–1.14
1001–1500	1530	0.91	0.71–1.12
1501–2000	1161	1.10	0.79–1.35
≥2001	1306	1.34	1.02–1.73

GCS: Glasgow Coma Scale. ISS: Injury Severity Score. NISS: New Injury Severity Score. AIS: Abbreviated Injury Scale.

**Table 6:** Multivariate regressions analysis in patients after severe trauma, a subgroup analysis in patients without severe traumatic brain injury.

Without AIS head ≥ 4; *n* = 4454	Odds ratio	95% CI
Age, y		
0–54	Reference	
55–64	2.09	1.39–3.16
65–74	7.69	5.37–11.02
≥75	23.13	16.04–33.36
Revised Trauma Score		
GCS 13–15	Reference	
GCS 9–12	1.55	1.09–2.24
GCS 8–6	1.91	1.21–3.01
GCS 5-4	4.24	2.24–8.01
GCS 3	3.31	2.20–4.97
Prehospital blood pressure, mmHg		
≥91	Reference	
61–90	1.93	1.44–2.59
0–60	3.66	2.31–5.81
Blunt trauma	Reference	
Penetrating trauma	1.26	0.72–2.19
Prehospital intubation	1.33	0.93–1.89
Prehospital catecholamines	1.91	1.33–2.76
Prehospital resuscitation	1.55	0.78–3.09
Prehospital chest tube	0.75	0.48–1.04
NISS	1.07	1.06–1.09
Prehospital volume, mL		
0–500	Reference	
501–1000	1.44	0.89–2.35
1001–1500	1.77	1.08–2.92
1501–2000	2.24	1.32–3.80
≥2001	2.71	1.62–4.52

GCS: Glasgow Coma Scale. ISS: Injury Severity Score. NISS: New Injury Severity Score. AIS: Abbreviated Injury Scale.

**Table 7:** Multivariate regressions analysis in patients after severe trauma, a subgroup analysis in patients with severe traumatic brain injury.

With AIS head ≥ 4; *n* = 3187	Odds ratio	95% CI
Age, y		
0–54	Reference	
55–64	1.68	1.25–2.28
65–74	2.85	2.17–3.74
≥75	7.53	5.71–9.92
Revised Trauma Score		
GCS 13–15	Reference	
GCS 9–12	1.44	1.02–2.03
GCS 8–6	1.89	1.34–2.67
GCS 5-4	3.18	2.15–4.72
GCS 3	4.96	3.58–6.88
Prehospital blood pressure mmHg		
≥91	Reference	
61–90	1.08	0.85–1.39
0–60	1.75	1.16–2.67
Blunt trauma	Reference	
Penetrating trauma	2.25	1.34–3.82
Prehospital intubation	1.41	1.03–1.92
Prehospital catecholamines	1.37	1.05–1.81
Prehospital resuscitation	2.25	1.37–3.62
Prehospital chest tube	0.98	0.67–1.43
NISS	1.05	1.04–1.05
Prehospital volume mL		
0–500	Reference	
501–1000	0.79	0.61–1.04
1001–1500	0.71	0.52–0.94
1501–2000	0.82	0.56–1.07
≥2001	1.12	0.72–1.39

GCS: Glasgow Coma Scale. ISS: Injury Severity Score. NISS: New Injury Severity Score. AIS: Abbreviated Injury Scale.

## References

[1] Hess J. R., Brohi K., Dutton R. P. (2008). The coagulopathy of trauma: a review of mechanisms. *Journal of Trauma*.

[2] Davis J. W., Hoyt D. B., McArdle M. S. (1992). An analysis of errors causing morbidity and mortality in a trauma system: a guide for quality improvement. *Journal of Trauma*.

[3] Gruen R. L., Jurkovich G. J., McIntyre L. K., Foy H. M., Maier R. V. (2006). Patterns of errors contributing to trauma mortality: lessons learned from 2,594 deaths. *Annals of Surgery*.

[4] Søreide K., Krüger A. J., Vårdal A. L., Ellingsen C. L., Søreide E., Lossius H. M. (2007). Epidemiology and contemporary patterns of trauma deaths: changing place, similar pace, older face. *World Journal of Surgery*.

[5] Lendemans S., Kreuzfelder E., Waydhas C., Nast-Kolb D., Flohé S. (2004). Clinical course and prognostic significance of immunological and functional parameters after severe trauma. *Unfallchirurg*.

[6] Sauaia A., Moore F. A., Moore E. E. (1995). Epidemiology of trauma deaths: a reassessment. *Journal of Trauma*.

[7] American College of Surgeons Committee on Trauma (2008). *ATLS Student Course Manual*.

[8] Bickell W. H., Stern S. (1998). Fluid replacement for hypotensive injury victims: how, when and what risks?. *Current Opinion in Anesthesiology*.

[9] Bickell W. H., Barrett S. M., Romine-Jenkins M., Hull S. S., Kinasewitz G. T. (1994). Resuscitation of canine hemorrhagic hypotension with large-volume isotonic crystalloid: impact on lung water, venous admixture, and systemic arterial oxygen saturation. *The American Journal of Emergency Medicine*.

[10] Bickell W. H. (1993). Are victims of injury sometimes victimized by attempts at fluid resuscitation?. *Annals of Emergency Medicine*.

[11] Branas C. C., Sing R. F., Davidson S. J. (1995). Urban trauma transport of assaulted patients using nonmedical personnel. *Academic Emergency Medicine*.

[12] Dalton A. M. (1995). Prehospital intravenous fluid replacement in trauma: an outmoded concept?. *Journal of the Royal Society of Medicine*.

[13] Kaweski S. M., Sise M. J., Virgilio R. W. (1990). The effect of prehospital fluids on survival in trauma patients. *Journal of Trauma*.

[14] Dula D. J., Wood G. C., Rejmer A. R., Starr M., Leicht M. (2002). Use of prehospital fluids in hypotensive blunt trauma patients. *Prehospital Emergency Care*.

[15] Talving P., Pålstedt J., Riddez L. (2005). Prehospital management and fluid resuscitation in hypotensive trauma patients admitted to Karolinska University Hospital in Stockholm. *Prehospital and Disaster Medicine*.

[16] Humann B., Taeger G., Lefering R. (2011). Lethality and outcome in multiple injured patients after severe abdominal and pelvic trauma: influence of preclinical volume replacement—an analysis of 604 patients from the trauma registry of the DGU. *Unfallchirurg*.

[17] Hussmann B., Lefering R., Waydhas C. (2013). Does increased prehospital replacement volume lead to a poor clinical course and an increased mortality? A matched-pair analysis of 1896 patients of the Trauma Registry of the German Society for Trauma Surgery who were managed by an emergency doctor at the accident site. *Injury*.

[18] Hußmann B., Lefering R., Taeger G. (2011). Influence of prehospital fluid resuscitation on patients with multiple injuries in hemorrhagic shock in patients from the DGU trauma registry. *Journal of Emergencies, Trauma and Shock*.

[19] Hussmann B., Lefering R., Kauther M. D., Ruchholtz S., Moldzio P., Lendemans S. (2012). Influence of prehospital volume replacement on outcome in polytraumatized children. *Critical Care*.

[20] Haut E. R., Kalish B. T., Cotton B. A. (2011). Prehospital intravenous fluid administration is associated with higher mortality in trauma patients: a national trauma data bank analysis. *Annals of Surgery*.

[21] O'Gorman M., Trabulsy P., Pilcher D. B. (1989). Zero-time prehospital IV. *Journal of Trauma*.

[22] Slovis C. M., Herr E. W., Londorf D., Little T. D., Alexander B. R., Guthmann R. J. (1990). Success rates for initiation of intravenous therapy en route by prehospital care providers. *The American Journal of Emergency Medicine*.

[23] Clarke J. R., Trooskin S. Z., Doshi P. J., Greenwald L., Mode C. J. (2002). Time to laparotomy for intra-abdominal bleeding from trauma does affect survival for delays up to 90 minutes. *Journal of Trauma—Injury, Infection and Critical Care*.

[24] Soudry E., Stein M. (2004). Prehospital management of uncontrolled bleeding in trauma patients: nearing the light at the end of the tunnel. *Israel Medical Association Journal*.

[25] Tan P. G., Cincotta M., Clavisi O. (2011). Review article: prehospital fluid management in traumatic brain injury. *Emergency Medicine Australasia*.

[26] Levy M. M., Fink M. P., Marshall J. C. (2003). 2001 SCCM/ESICM/ACCP/ATS/SIS international sepsis definitions conference. *Critical Care Medicine*.

[27] Vincent J.-L., Moreno R., Takala J. (1996). The SOFA (Sepsis-related Organ Failure Assessment) score to describe organ dysfunction/failure. On behalf of the Working Group on Sepsis-Related Problems of the European Society of Intensive Care Medicine. *Intensive Care Medicine*.

[28] Pepe P. E., Mosesso V. N., Falk J. L. (2002). Prehospital fluid resuscitation of the patient with major trauma. *Prehospital Emergency Care*.

[29] Kowalenko T., Stern S., Dronen S., Wang X. (1992). Improved outcome with hypotensive resuscitation of uncontrolled hemorrhagic shock in a swine model. *Journal of Trauma*.

[30] Bickell W. H., Bruttig S. P., Millnamow G. A., O'Benar J., Wade C. E. (1991). The detrimental effects of intravenous crystalloid after aortotomy in swine. *Surgery*.

[31] Riddez L., Johnson L., Hahn R. G. (1998). Central and regional hemodynamics during crystalloid fluid therapy after uncontrolled intra-abdominal bleeding. *Journal of Trauma—Injury, Infection and Critical Care*.

[32] Geeraedts L. M. G., Kaasjager H. A. H., van Vugt A. B., Frölke J. P. M. (2009). Exsanguination in trauma: a review of diagnostics and treatment options. *Injury*.

[33] Demetriades D., Chan L., Cornwell E. (1996). Paramedic vs private transportation of trauma patients: effect on outcome. *Archives of Surgery*.

[34] Cornwell E. E., Belzberg H., Hennigan K. (2000). Emergency medical services (EMS) vs non-EMS transport of critically injured patients: a prospective evaluation. *Archives of Surgery*.

[35] Sloan E. P., Koenigsberg M., Weir W. B. (2015). Emergency resuscitation of patients enrolled in the US diaspirin cross-linked hemoglobin (DCLHb) clinical efficacy trial. *Prehospital and Disaster Medicine*.

[36] Turner J., Nicholl J., Webber L., Cox H., Dixon S., Yates D. (2000). A randomised controlled trial of prehospital intravenous fluid replacement therapy in serious trauma. *Health Technology Assessment*.

[37] Bratton S. L., Chestnut R. M., Ghajar J. (2007). Guidelines for the management of severe traumatic brain injury. I. Blood pressure and oxygenation. *Journal of Neurotrauma*.

[38] Holcomb J. B. (2003). Fluid resuscitation in modern combat casualty care: lessons learned from Somalia. *Journal of Trauma—Injury, Infection and Critical Care*.

